# Performance of Endophyte Infected Tall Fescue in Europe and North America

**DOI:** 10.1371/journal.pone.0157382

**Published:** 2016-06-10

**Authors:** Kari Saikkonen, Timothy D. Phillips, Stanley H. Faeth, Rebecca L. McCulley, Irma Saloniemi, Marjo Helander

**Affiliations:** 1 Natural Resources Institute Finland (Luke), Turku, Finland; 2 Department of Plant and Soil Sciences, University of Kentucky, Lexington, Kentucky, United States of America; 3 Department of Biology, University of North Carolina, Greensboro, North Carolina, United States of America; 4 Department of Biology, University of Turku, Turku, Finland; Wageningen University and Research Centre, NETHERLANDS

## Abstract

Human assisted plant invasions from Europe to North America have been more common than the reverse. We tested endophyte-mediated performance of tall fescue in parallel three year experiments in Europe and the USA using endophyte infected and uninfected wild and cultivated plants. Experimental plants were subjected to nutrient and water treatments. Whereas endophyte infection increased tall fescue performance in general, the effects of endophytes on plant growth and reproduction varied among plant origins under different environmental conditions. Naturally endophyte-free Finnish cultivar ‘Retu’ performed equally well as ‘Kentucky-31’ in both geographic locations. All Eurasian origin plants performed well in the US. In Finland, plants established well and both cultivars survived over the first winter. However, winter mortality of ‘Kentucky-31’ plants was higher, particularly in fertilized soils in the subsequent winters. Our results suggest that tall fescue ecotype ‘Kentucky-31’ that flourishes in North America is poorly adapted to Northern European conditions.

## Introduction

Successful plant invasions have been largely unidirectional from Europe to North America since the last Ice Age [1–4]. Since European colonists discovered the Americas, human migration and increased global trade have facilitated species interchange between the Old World and the Western Hemisphere, via deliberate introduction of crop and ornamental plants and unintentional introductions, such as stowaways. Human-assisted species introductions fail, however, to explain successful naturalization of Eurasian plant species into America in general. The successful invasion and subsequent naturalization of alien species is often linked to the characteristics of the invading species such as self-fertility, reproductive strategies, vigor, phenotypic plasticity, and genetic variation that allows passage through abiotic and biotic environmental filters and provides higher potential for rapid adaptive evolution to new conditions. In contrast, the lower probability of colonization of North American plants to Europe may be at least partially explained by maladaptation of North American plants to comparable climate zones in Western Europe that have different photoperiods. In general, climates in Western Europe that are similar to North American climates are at higher latitudes and thus have greater seasonal changes in photoperiod [5–8,4].

The invasiveness of plant species may also be tightly linked with presence of mutualistic symbionts and other service providers such as pollinators, or empty niche and/or enemy escape in the recipient environment [9–15]. For example, ectomycorrhizal fungi are vitally important for the distribution of approximately 6000 tree species including many dominant and economically important species near subarctic tree-lines [16–18]. The importance of microbes to plant community dynamics is, however, context dependent, incorporating multispecies interactions and community feedbacks, modifying niches of species competing and/or sharing common resources [19].

Temperate grasslands are particularly vulnerable to plant invasions because they have a long history of being driven by human activities such as agriculture [20,21]. In North America, European settlers opened windows for plant invasions by rapidly converting native grasslands and forests into arable lands and pastures. The concomitant species introductions were largely unidirectional from Europe to North America. The great success of many European species in North America may have been facilitated by plant species adaptations to heavy grazing by cattle due to their long-term coexistence with humans in the Eurasian environment from whence they originated [3,20]. Before human settlers, the Holocene North America grasslands had primarily been structured and maintained by fires. In the intermountain west and Great Plains, grazing by bison also maintained grasslands. However, large areas of North American grasslands in eastern North America evolved under the lack of heavy gazing pressure until humans started to regulate fires, reintroduced the horse, and introduced cattle in the 1600s [3]. Increasing agriculture in conjunction with these other anthropogenic changes in type, frequency and intensity of disturbances greatly facilitated the establishment and naturalization of nonnative plant species into grasslands and destruction of native grassland plant communities in North America [20,22].

Many grass species were accidentally or intentionally introduced into N. America, the latter because of their agronomic value and high tolerance of a wide range of biotic and abiotic conditions. Alien grasses have become tenacious invaders of Native American grassland communities. Increasing evidence suggests that the success of some of the alien cool season grasses may be due, at least in part, to their asymptomatic and systemic endophytic fungi [11]. Tall fescue [*Schedonorus phoenix* (Scop.) Holub. ex. *Lolium arundinaceum*, syn. *Festuca arundinacea*] put these systemic grass-endophyte symbioses on the map in the 1970s when livestock disorders associated with the recently commercialized tall fescue cultivar ‘Kentucky 31’ (KY-31) were attributed to mycotoxins produced by the symbiotic *Epichloë* endophyte [23–26]. Tall fescue was originally introduced from Europe in the late 1800’s, probably as a contaminant in hay or packing materials, and now it is the most important cool-season grass in the United States [27,28]. The obligately outbreeding allohexaploid cultivar KY-31, which is commonly infected with *Epichloë coenophiala* (Morgan-Jones & W. Gams) C.W. Bacon & Schardl (ex. *Neotyphodium coenophialum* Glenn, Hanlin & Bacon) endophyte, is adapted to a wide range of soil types, fertility and pH, and tolerant to moderate cold and heat stress. It has been and is still widely used for animal feed, lawns and turf, soil stabilization and wildlife food plots in humid areas of the US. In 1977, KY-31 tall fescue composed approximately 97% of all tall fescue turf in the United States [29]. Similar to other endophyte-grass symbiota, endophyte infection frequency in tall fescue varies, but regional and herbarium studies have reported that on average 60–80% of individuals are infected [30,31]. Since then, this highly vigorous cultivar has become a tenacious invader of managed and unmanaged grasslands threatening the persistence of native plant species diversity throughout the much of the eastern United States [11,27,28,30].

The competitive superiority of tall fescue is promoted by *E*. *coenophiala* endophyte [11] particularly in high nutrient agro-environments [32]. Before the European settlers, only part of the eastern United States was heavily grazed by native vertebrate grazers [3,20]. In contrast, the present tall fescue green belt is under high grazing pressure by cattle [27], and accordingly the anti-herbivore advantage provided by the endophyte to the host has been suggested to cause a rapid increase in endophyte frequency in host populations [30]. Because these grass endophytes are vertically transmitted from maternal plant to offspring via seeds, the invasive grass host is a phenotypic combination of plant and microbe with a long co-evolutionary history. In this symbiosis, the host plant often receives increased growth and reproduction, and protection from pathogens and herbivores provided by fungal produced alkaloids [33–39], while the plant serves as a shelter, nutrient provider and transmission aid to the fungus. Thus, grass endophyte symbiosis is often considered to be mutualistic [11,25].

However, the empirical evidence supporting the purported grass-endophyte mutualism has historically been dominated by a few agronomic and non-native model systems [38,39], particularly the tall fescue cultivar KY-31 and its endophytic partner *E*. *coenophiala* in the US. In contrast, although this grass species is widely distributed in Europe, infected grasses do not deter animal grazing nor are they competitively dominant in native or human-modified ecosystems [31,32]. Tall fescue is described as a species complex consisting of three major (Continental, Mediterranean and rhizomatous) morphotypes [40,41]. The Continental morphotype is spread over northern Europe and was also the germplasm stock for the cool-season cultivars in the US, including KY-31. Today, tall fescue is an increasingly important forage grass also throughout Europe, and many cultivars are commonly used in agriculture.

In this study, we examined if the success of tall fescue in the US is specific to host population-level genotypic characteristics, endophyte infection, or high nutrient agro-environments. Specifically we studied in parallel experiments in Finland and in the US how endophyte infection, plant origin and nutrient and water availability affect the growth and reproductive capacity of tall fescue. The experiment in Finland was situated in a boreal climate at the same latitude with Fairbanks, Alaska, whereas the experiment in North America is situated in the temperate zone at the same latitude with Athens, Greece in southernmost Europe. In addition to wild plants collected from three geographic locations in the northernmost distribution range of the species in Europe, we used two cultivars (KY-31 and ‘Retu’ from US and Europe, respectively) in these experiments. First, we predicted that plants would perform best in the climatic and latitudinal environment to which they were adapted. Second, we hypothesized that variation in performance would be lower in cultivars compared to grasses from wild populations because of lower genetic variation due to breeding. Consequently, the adaptive capacity and performance of cultivars across a wide range of enviroments should be lower as well. In addition to plant origin, we predicted that plant performance would be affected by the symbiosis with *Epichloë* endophytes. Past literature suggests that the mutualism between *E*. *coenophiala* and KY-31 is strongest in high nutrient agro-environments and may be atypical of other endophyte-tall fescue interactions [38,39,42]. Thus, we predicted that compared to KY-31, the benefits from endophyte infection would be lesser in wild grasses evolved under low herbivory pressure but instead may depend more heavily on nutrient availability in soils.

## Materials and Methods

### Plant material

In August 2005, we collected seeds from natural tall fescue populations at three geographic locations in the Baltic Sea that were isolated by approx. 500 km from each other: the island of Åland (A) (8 populations), island of Gotland (G) (9 populations) and west coast of Sweden (S) (6 populations). We collected 10 to 50 plant individuals from each population. The field studies did not involve endangered or protected species and public right of access in Nordic countries allowed us to collect the samples without specific permissions. The presence/absence of systemic endophyte infection was checked by microscopic examination of three seeds from each individual plant [43]. All the studied tall fescue populations had seed borne *Epichloë coenophiala* infections varying in infection frequency from 85–100% [31]. We combined all naturally E- (uninfected) and E+ (infected) seeds separately from each of the three geographic origins (Åland, Gotland and Sweden). In addition to plants from natural tall fescue populations, both E+ and E- ‘Kentuky 31’ cultivar (KY-31) seeds were obtained from the University of Kentucky, as were E- Finnish tall fescue cultivar ‘Retu’ (R) seeds from the Plant Inspection Centre, Seed Testing Department, Loimaa, Finland (www.evira.fi). Identical sets of seeds were used in parallel field experiments in Finland and USA.

### Endophyte removal

A subset of the naturally endophyte infected (E+) seeds were heat-treated by soaking them in warm water (56–57°C) for 10–20 minutes to kill the fungus while the seed remained viable. Using these manipulatively endophyte-free (ME-) plants, we aimed to separate the effects of endophyte from the phenotypic responses of the endophyte-grass symbiotum, and/or estimate if the plant partner is adapted to the symbiosis during a coevolutionary relationship [39]. We assume that losing the long-time mutualistic fungal partner would lead to lower performance of ME- plants than naturally endophyte-free (E-) plants in the same environment.

### Parallel field experiments in Finland and USA

The two parallel common garden experiments were carried out in the fields of Turku Botanical Garden, University of Turku, Finland (60°26′0″N, 22°10′19″E) and at the University of Kentucky experimental farm in Eden Shale, Kentucky, USA (38°32’22”N, 84°44’24”W). The field site in Finland is at the edge of the northern distribution range of natural tall fescue populations, while the Kentucky field site is situated in the heart of the intensive tall fescue cultivation area in USA–also known as the fescue belt (http://forages.oregonstate.edu/tallfescuemonograph/) and not far from where the tall fescue that was eventually developed into the cultivar KY-31 was originally introduced in the 1880’s (near Frenchburg, Kentucky). Both experimental field sites had been in cultivation in the past and were tilled without nutrient enrichment in the summer 2004. Winter temperatures were lower in Finland and precipitation higher in Kentucky (S1 Fig). Although winters in Finland are characterized by months-long frost temperatures, greater snow cover may provide better protection for plants in the winter compared to Kentucky. According to the Finnish Meteorological Institute's measurements (Finnish Meteorological Institute http://www.fmi.fi/en), in 2006 the thermal growing season was slightly longer than usual, and temperatures were higher. The sum of effective temperature (the sum of the positive differences between diurnal mean temperatures and 5°C) reached new records, and consequently the summer 2006 was the driest ever experienced in Southern Finland.

The experimental set-up was a randomized block design consisting of 10 blocks, each including 4 plots. In each of these 40 plots we planted grasses from following geographic origin—endophyte combinations: natural populations [Åland (A), Gotland (G) and coastal Sweden (S)], cultivar [‘Kentucky 31’ (KY-31)] and endophyte infection statuses (E+, E-, ME-), and endophyte-free (E-) cultivar ‘Retu’ (R). In every plot, we had one plant from each origin and endophyte status. Because cultivar ‘Retu’ was always endophyte free (E-), and in the Kentucky experiment we had only E+ and E- plants of KY-31-origin, there were 13 and 12 plants in each plot in Finland and in Kentucky, respectively. The 40 plots consisting of 13 or 12 plants summed up to total of 520 plants in Finland and 480 plants in Kentucky. Because naturally occurring and agronomic fields of fescue are never single plant genotypes, we chose a population-level approach to this study and did not quantify individual plant genotype responses to our treatments.

The tall fescue seeds were germinated on moist tissue paper in Petri dishes in a greenhouse and planted into individual pots with sand and peat mixture 7 days after germination. Plants were grown in the greenhouse until they had 3 tillers and were then planted to the respective fields about 0.5 m apart from each other and from the edge of the plot in August 2004.

The four plots in each block were randomly designated to one of the four treatments: control (C), water (W), nutrient (N), and combined water and nutrient (WN). The control treatment received only ambient rainfall. In Finland, W treatment plots received 3 L of water applied to each plant separately three times a week from June to August, for N treatment plants 50 g of granular N-P-K-fertilizer (Nurmen Y2, Kemira KnowHow, N-P-K/20-6-6) was applied two times during the growing seasons, and WN treatment received both water and nutrient applications. In Kentucky, W treatment plants received 3.8 L of water twice a week from April to October, and fertilization consisted of 50 kg/ha of N per application as urea. The amount of water applied corresponds to 350 mm precipitation which doubled the amount of water received by plants during the annual treatment period in Kentucky in both years and in Finland in 2005. In Finland, the W treatment quadrupled the amount of water received in the exceptionally dry summer of 2006. We acknowledge that numerous uncontrolled differences between the two experimental sites may confound the comparison of interpretations from the two experiments. To take into account the differences in nutrient contents in soils, we analysed soil samples from the untreated (control) experimental plots. The soil pH was 6.7 and 5.9, total nitrogen 0.15% and 0.14%, phosphorus 7 and 14 mg/kg, potassium 132 and 81 mg/kg, calcium 1800 and 2080 mg/kg and magnesium 208 and 120 mg/kg for Finland and Kentucky sites, respectively. The water and nutrient treatments were applied to the experiments in two growing seasons (2005 and 2006).

The experimental areas were fenced to prevent large vertebrates (e.g., rabbits, deer) from disturbing the experimental plants. However, smaller vertebrates (e.g., voles) and invertebrates were able to freely access the area. The space between the experimental plants was either hand weeded or sprayed with herbicide (glyphosate Roundup®Bio) two times during the growing season to prevent interspecific competition between weeds and the experimental plants.

### Response variables

In 2005, all the experimental plants were double-checked to verify their endophyte status. First, we sampled a pseudostem from each plant for immunoblot assay to detect monoclonal antibodies specific to *Epichloë* (Phytoscreen Immunoplot Kit #ENDO7973, Agrinostics, Watkinsville, Georgia, USA). In addition, at the end of the summer, three seeds from each plant were stained [43] and endophyte infections were checked by microscopic examination. The endophyte status of four and 35 plants out of 520 and 480 plants were reassigned to a correct endophyte status in the Finland and Kentucky gardens, respectively.

The survival of the experimental plants was recorded during the three study years (2005–2007). The vegetative growth and reproductive allocation of the plants was observed in 2005 and 2006 at both study sites at the end of growing season: the number of flowerheads was counted on a per plant basis and the above ground biomass of individual plants was harvested by cutting the tillers at the height of 5 cm above the ground using a rice cutting sickle. The biomass was then dried and weighed.

### Statistical analyses

All the statistical analyses were performed in the R environment (R Core Team 2012). The mortality of plants was analysed using general linear model glm in R [44] with binomial distribution and logit-link function. The statistical model for survival included endophyte status, nutrient treatments (N: N and WN combined) and control treatments (C: C and W combined), and plant origins (cultivar ‘Retu’ as baseline, KY-31, A, G, S) and their interactions.

Plant biomass and flowerhead counts were analyzed using general linear mixed models program lme in R. The model included study year (2005, 2006), country (Finland, Kentucky), endophyte infection status (E+, E-, ME-), grass origin (KY-31, Retu, A, G, S), treatments (C, W, N, WN) as fixed factors and block as a random factor. Normality of biomass and flowerhead counts was gained after square root transformation (as suggested by the Box-Cox analysis, *ℓ* = ½).

## Results

### Survival

**Fig 1:**
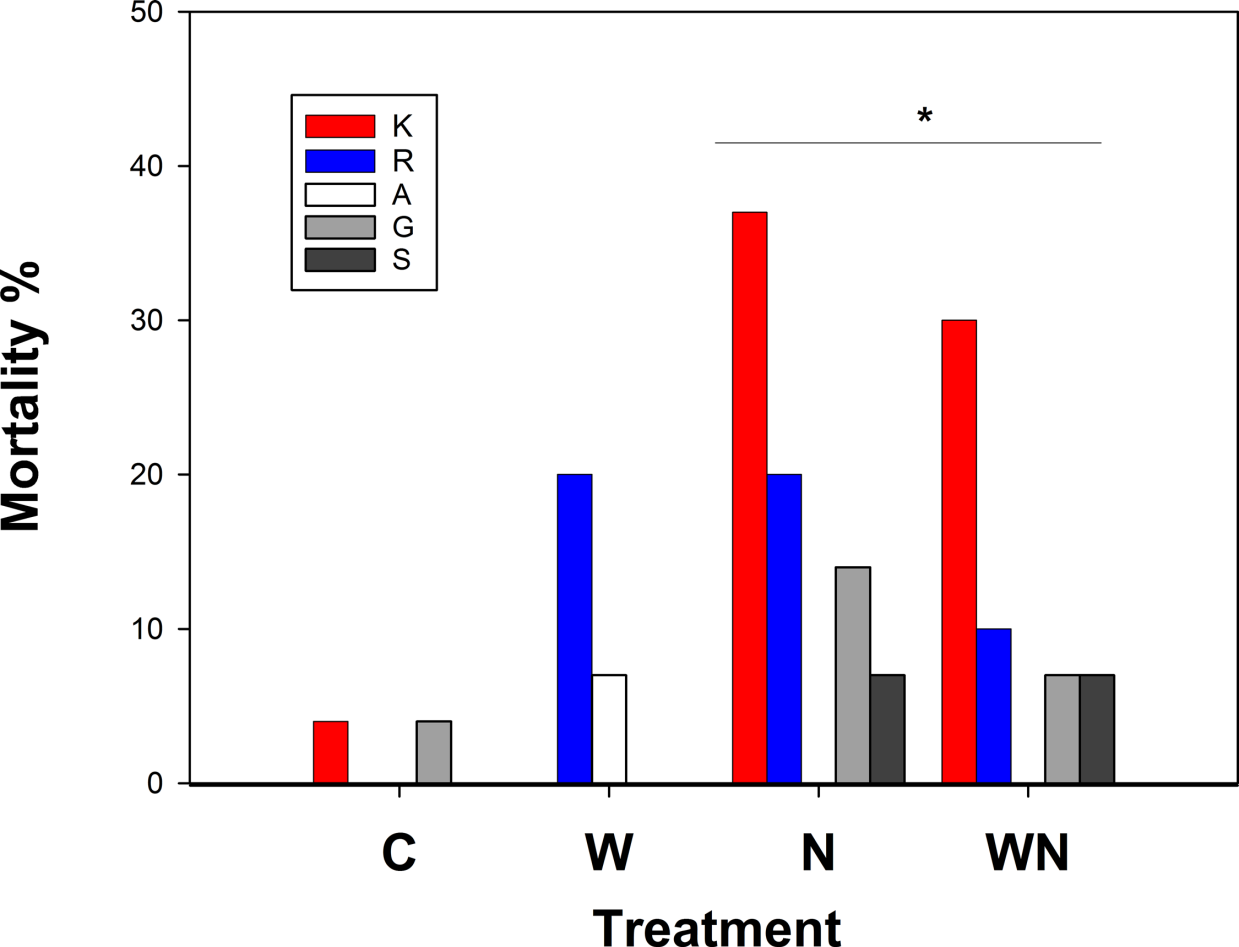
Mortality of tall fescue plants in Finland in the end of the experiment. Plant origins: K = ‘Kentucky 31’ cultivar, R = ‘Retu’ cultivar, A = wild type from Åland, G = wild type from Gotland, S = wild type from costal Sweden. Treatments: C = control, W = water application, N = nutrient application, WN = water and nutrient application. The origin-treatment interaction is indicated by * (see Table 1).

Plant survival was higher in Kentucky than in Finland. None of the plants died in Kentucky, whilst in Finland the death rate gradually increased from one plant in the first winter to 21 and 19 plants in the following two winters. In Finland survival was lowest in KY-31 (83%), while the other origins ranged from 88% (Retu) to 98% (A) (comparison between origins: n = 520, *x*^2^ = 28.1, df = 4, p<0.0001; Fig 1). Mortality was higher in nitrogen fertilized plants (12.7% vs. 2.3%, n = 520, *x*^2^ = 18.7, df = 1, p<0.0001) regardless of the endophyte infection status of the plant (survivals vs endophyte: *x*^2^ = 0.16, df = 2, p = 0.92). In the logit model, survival of fertilized KY-31 plants in Finland (interaction N*KY-31: z = 2.05, P <0.04) was lowest, but in other origins fertilization did not affect survival (Table 1).

**Table 1:** Mortality of tall fescue plants in Finland.

	Estimate	z	p	
**Intercept**	2,20	2.948	0.0032	**
**Orig K**	1,88	1.499	0.1338	
**Orig A**	1,17	1.130	0.2586	
**Orig G**	1,88	1.499	0.1338	
**Orig S**	17,37	0.013	0.9900	
**Treat N**	-0,46	-0.475	0.6346	
**Orig K: Treat N**	-2,92	-2.046	0.0408	*
**Orig A: Treat N**	16,66	0.012	0.9904	
**Orig G: Treat N**	-1,42	-0.967	0.3336	
**Orig S: Treat N**	-16,46	-0.012	0.9905	

Logit model for mortality in Finland as a function of origin (Orig) (three wild populations and two cultivars: A = Åland island, G = Gotland island, S = coastal Sweden, K = cultivar ‘Kentucky-31’, Retu = cultivar ‘Retu’) and treatments (Treat) (Nutrient treatments N = N and WN combined, control treatments C = C and W combined) and their interaction. The Finnish cultivar ‘Retu’ without fertilization was used as the baseline for comparisons. Model estimates and Wald statistics (Z) with p-values (** < 0.01,* < 0.05, o < 0.1) are shown.

### Growth and reproduction

**Fig 2:**
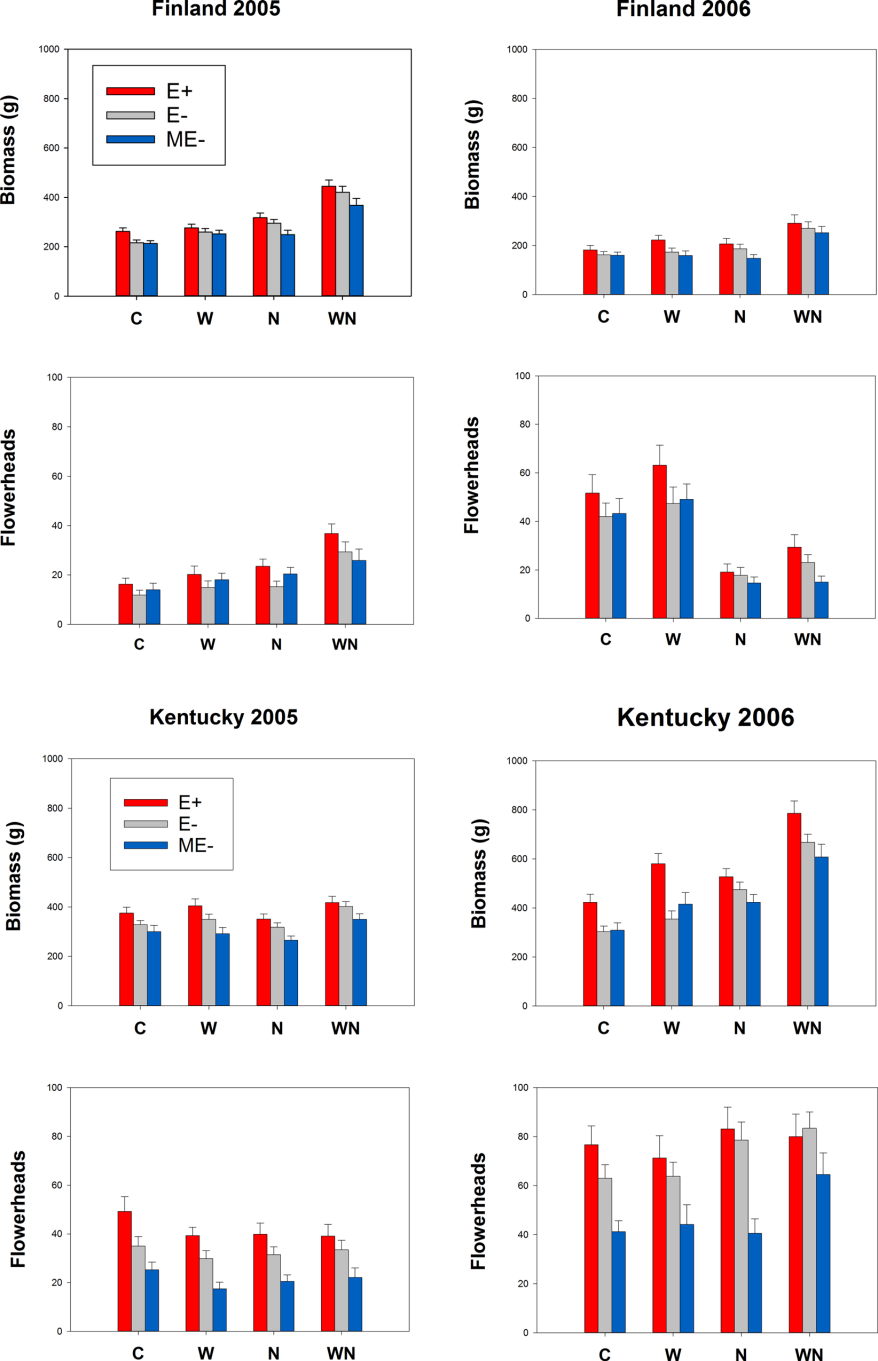
Growth and reproduction of endophyte-infected and endophyte-free tall fescue in different environmental conditions. Biomass (x±S.E.) and number of flowerheads (x±S.E.) of endophyte free (E-), endophyte infected (E+) and manipulatively endophyte free (ME-) tall fescue plants in Finland and Kentucky field experiments in years 2005 and 2006. The plants received either only ambient rain (C) or water (W), nutrient (N) and combined water and nutrient (WN) treatments.

**Fig 3:**
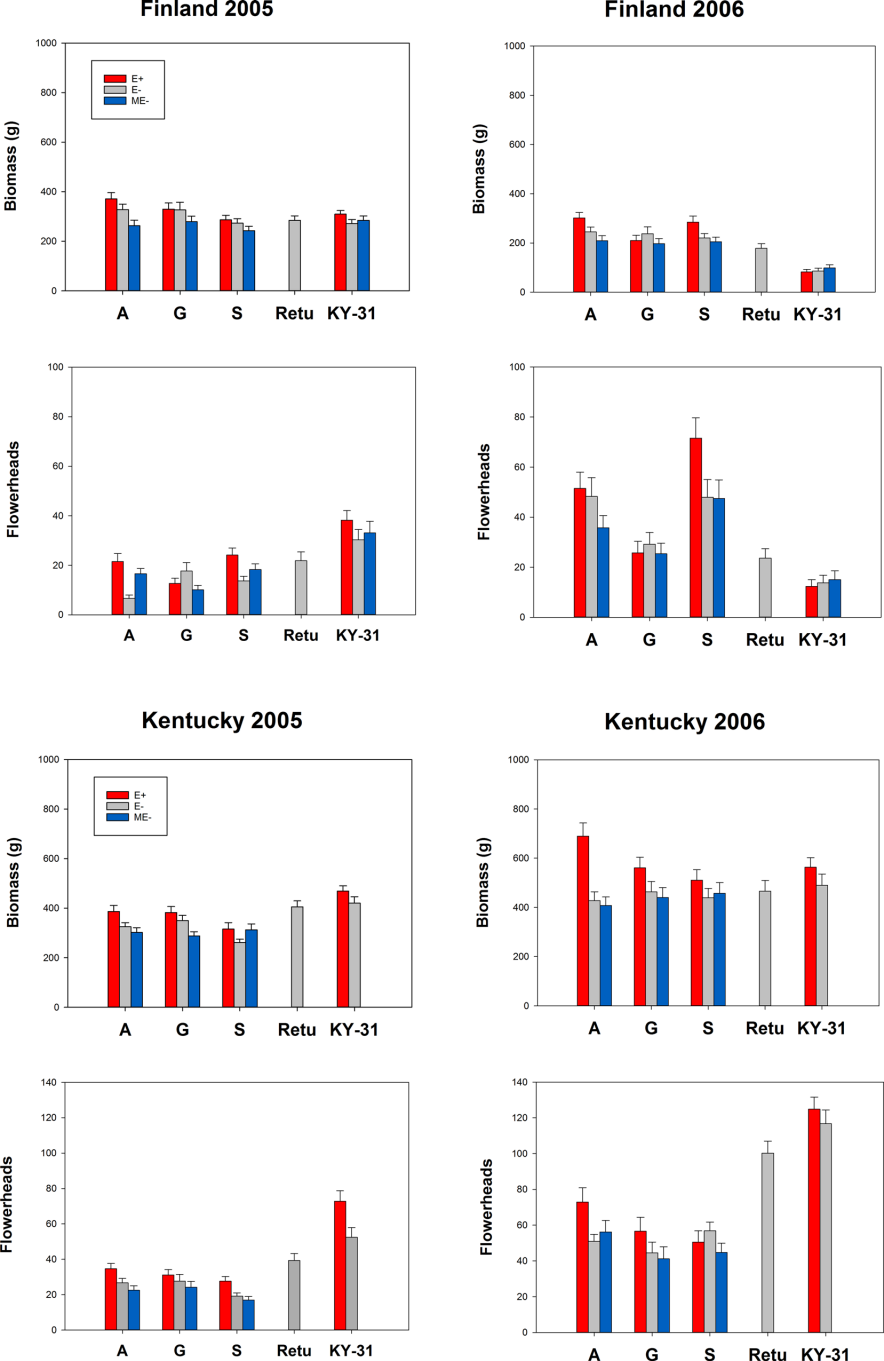
Growth and reproduction of endophyte-infected and endophyte-free tall fescue from different origins. Biomass (x±S.E.) and number of flowerheads (x±S.E.) of endophyte free (E-), endophyte infected (E+) and manipulatively endophyte free (ME-) tall fescue plants in Finland and Kentucky field experiments in years 2005 and 2006. Plants were collected from wild populations in Europe (A = island of Åland, G = Island of Gotland, S = coastal Sweden) or were cultivars from Europe (Retu) or USA (KY-31).

Plant growth and reproduction differed between the two geographic locations and study years (Figs 2 and 3, Tables 2 and 3, S2 Fig). During the first growing season (2005), the plants in Finland were smaller and produced fewer flowerheads (biomass mean ± S.E.: 297 ± 6, n = 506; flowerheads mean ± S.E.: 20 ± 1, n = 518) compared to plants in Kentucky (biomass mean ± S.E.: 353 ± 7, n = 480; flowerheads mean ± S.E.: 33 ± 1, n = 480). The difference was even more striking in the following year (2006), when the plants in Kentucky grew 60% larger and produced twice as many flowerheads compared to the plants in Finland (USA: biomass mean ± S.E.: 496 ± 13, n = 477; flowerheads mean ± S.E.: 69± 2, n = 456; Finland: biomass mean ± S.E.: 201 ± 6, n = 486; flowerheads mean ± S.E.: 35 ± 2, n = 497). It is noteworthy that in Finland in the exceptionally dry summer of 2006 (S1 Fig; Finnish Meteorological Institute http://www.fmi.fi/en), some of the KY-31 plants had died after harsh winter conditions, and some of the surviving KY-31 plants grew poorly.

**Table 2:** Linear mixed models for tall fescue biomass in Finland and Kentucky experiments in years 2005 and 2006.

	FINLAND						KENTUCKY					
	Estimate	S.E.	df	t	p		Estimate	S.E.	df	t	p	
**Intercept**	1203.51	1531.46	905	0.79	0.432		14324.53	1084.90	963	13.20	0.000	***
**Year**	-0.59	0.76	905	-0.77	0.439		-7.14	0.5410	963	-13.19	0.000	***
**E+**	2.03	0.35	905	5.73	0.000	***	0.73	0.29	963	2.46	0.014	**
**ME-**	-0.12	0.41	905	-0.29	0.775		-0.74	0.30	963	-2.49	0.013	**
**Treat N**	-8327.73	1165.44	905	-7.15	0.000	***	3169.91	934.96	963	3.39	0.001	***
**Treat W**	1.45	0.7925	905	1.83	0.068	o	-0.08	0.54	963	-0.15	0.884	
**Orig R**	1284.13	2442.35	905	0.53	0.599		-7448.68	1967.78	963	-3.79	0.000	***
**Orig A**	-4422.48	1829.99	905	-2.42	0.016	*	-11448.19	1378.63	963	-8.30	0.000	***
**Orig G**	-3978.66	1847.47	905	-2.15	0.032	*	-9292.29	1387.13	963	-6.70	0.000	***
**Orig S**	-5258.68	1833.22	905	-2.87	0.004	**	-13435.25	1390.16	963	-9.66	0.000	***
**Treat N:W**	0.77	0.60	905	1.29	0.197		2.31	0.47	963	4.97	0.000	***
**Treat W: Orig R**	0.92	1.78	905	0.75	0.451		-0.65	0.98	963	-0.66	0.509	
**Treat W: Orig A**	0.18	0.91	905	0.20	0.843		0.70	0.69	963	1.01	0.311	
**Treat W: Orig G**	0.77	0.92	905	0.84	0.403		1.91	0.69	963	2.77	0.006	**
**Treat W: Orig S**	1.09	0.91	905	1.20	0.232		1.24	0.69	963	1.79	0.073	o
**Year: Orig R**	-0.64	1.78	905	-0.53	0.599		3.72	0.98	963	3.79	0.000	***
**Year: Orig A**	2.21	0.91	905	2.42	0.016	*	5.71	0.69	963	8.31	0.000	***
**Year:Orig G**	1.98	0.92	905	2.15	0.032	*	4.63	0.69	963	6.70	0.000	***
**Year: Orig S**	2.62	0.91	905	2.87	0.004	**	6.70	0.69	963	9.67	0.000	***
**Year: Treat N**	4.15	0.58	905	7.15	0.000	***	-1.58	0.47	963	-3.39	0.001	***

**Fig 4:**
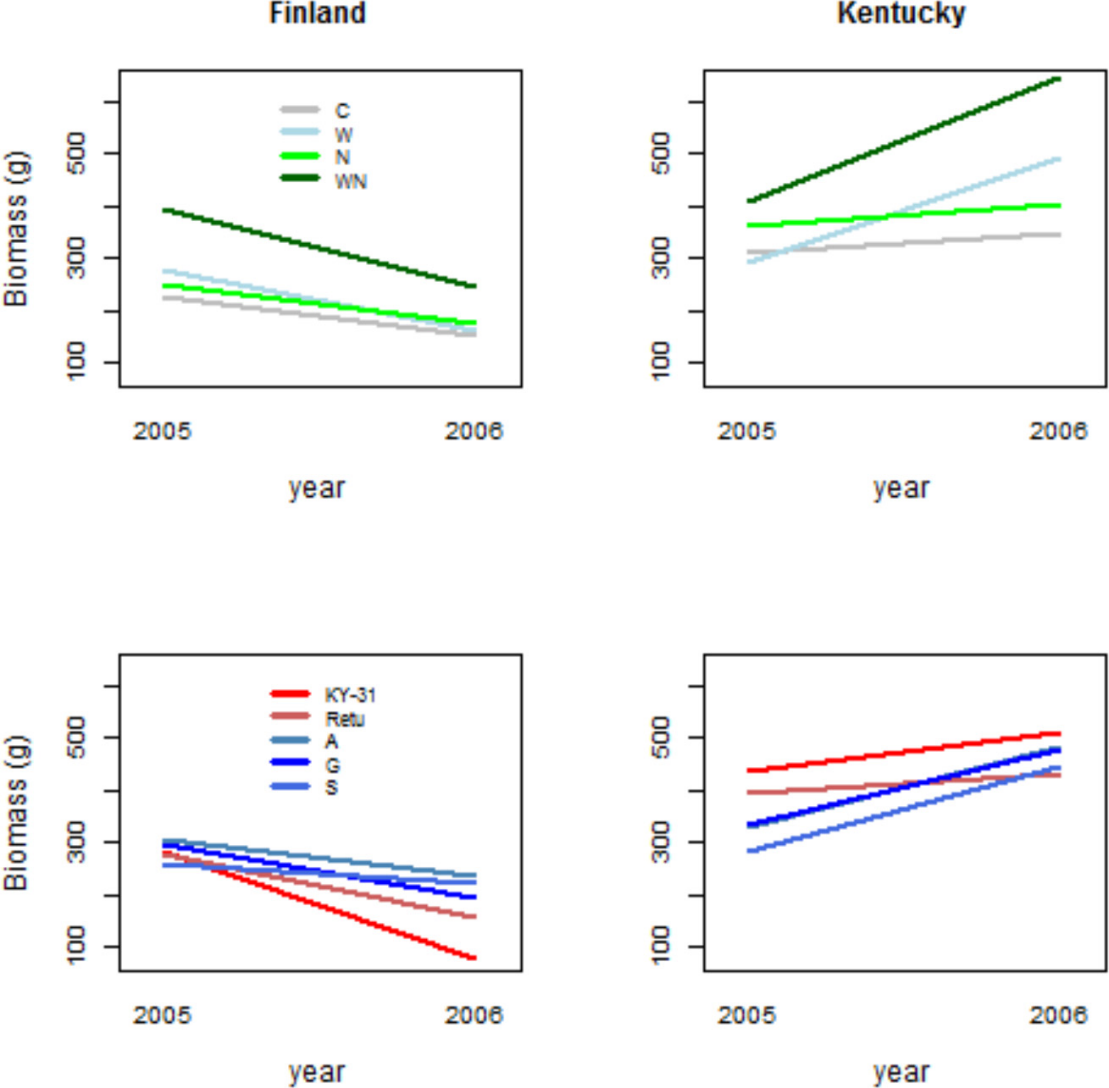
Effects of nutrient and water treatments, and plant origin on tall fescue growth. Model-based estimates for biomass (see Table 2 for model) on treatments (C = control, W = water treatment, N = nutrient treatment, WN = water and nutrient treatment) and three wild populations and two cultivars (A = Åland island, G = Gotland island, S = coastal Sweden, KY-31 = cultivar ‘Kentucky 31’, Retu = cultivar ‘Retu’) for tall fescue biomass in Finland and Kentucky experiments in the two study years, 2005 and 2006.

The plants were endophyte infected (E+; the baseline), endophyte free (E-) or manipulatively endophyte free (ME-), and they were given either water (W) nutrient (N) treatments or both (N:W) (Treat; C = control as the baseline). The grasses from the wild origins (Orig) Gotland (G), Åland (A) and Sweden (S) and cultivar ‘Retu’ (R) were compared with cultivar ‘Kentucky 31’ (KY-31; the baseline). Results for the same model are shown for both Finland and Kentucky experiment with all main factors and those interactions that were statistically significant in either of the experiments. The main interactions are shown graphically in Fig 4. Model estimates with standard errors (S.E.), degrees of freedom (df), and t-tests (t) with p-values (*** < 0.001,** < 0.01,* < 0.05, o < 0.1) are shown.

**Table 3:** Linear mixed models for tall fescue flowerheads in Finland and Kentucky experiments in years 2005 and 2006.

	FINLAND						KENTUCKY					
	Estimate	S.E.	df	t	p		Estimate	S.E.	df	t	p	
**Intercept**	-5381.64	745.76	909	-7.22	0.000	***	1652.51	648.91	967	2.55	0.011	*
**Year**	2.69	0.37	909	7.23	0.000	***	-0.82	0.32	967	-2.54	0.011	*
**E+**	0.59	0.17	909	3.48	0.001	***	0.63	0.18	967	3.55	0.000	***
**ME-**	-0.31	0.20	909	-1.54	0.125		0.03	0.18	967	0.19	0.847	
**Treat N**	-1862.97	567.53	909	-3.28	0.001	***	7229.67	559.18	967	12.93	0.000	***
**Treat W**	-0.33	0.22	909	-1.51	0.133		0.43	0.19	967	2.19	0.028	*
**Orig R**	-1378.57	1189.37	909	-1.16	0.247		-5613.80	1176.94	967	-4.77	0.000	***
**Orig A**	1646.34	891.14	909	1.85	0.065	o	-10572.12	824.54	967	-12.82	0.000	***
**Orig G**	3568.51	899.64	909	3.97	0.000	***	-7953.52	829.66	967	-9.59	0.000	***
**Orig S**	1725.19	892.69	909	1.93	0.054	o	-10935.16	831.50	967	-13.15	0.000	***
**Treat N:W**	0.54	0.29	909	1.86	0.063	o	0.49	0.28	967	1.75	0.081	o
**Year: Orig R**	0.69	0.59	909	1.16	0.247		2.80	0.59	967	4.77	0.000	***
**Year: Orig A**	-0.82	0.44	909	-1.85	0.065	o	5.27	0.41	967	12.82	0.000	***
**Year: Orig G**	-1.78	0.45	909	-3.97	0.000	***	3.97	0.41	967	9.59	0.000	***
**Year: Orig S**	-0.86	0.45	909	-1.94	0.053	o	5.45	0.41	967	13.15	0.000	***
**Year: Treat N**	0.93	0.28	909	3.28	0.001	**	-3.61	0.28	967	-12.93	0.000	***

**Fig 5:**
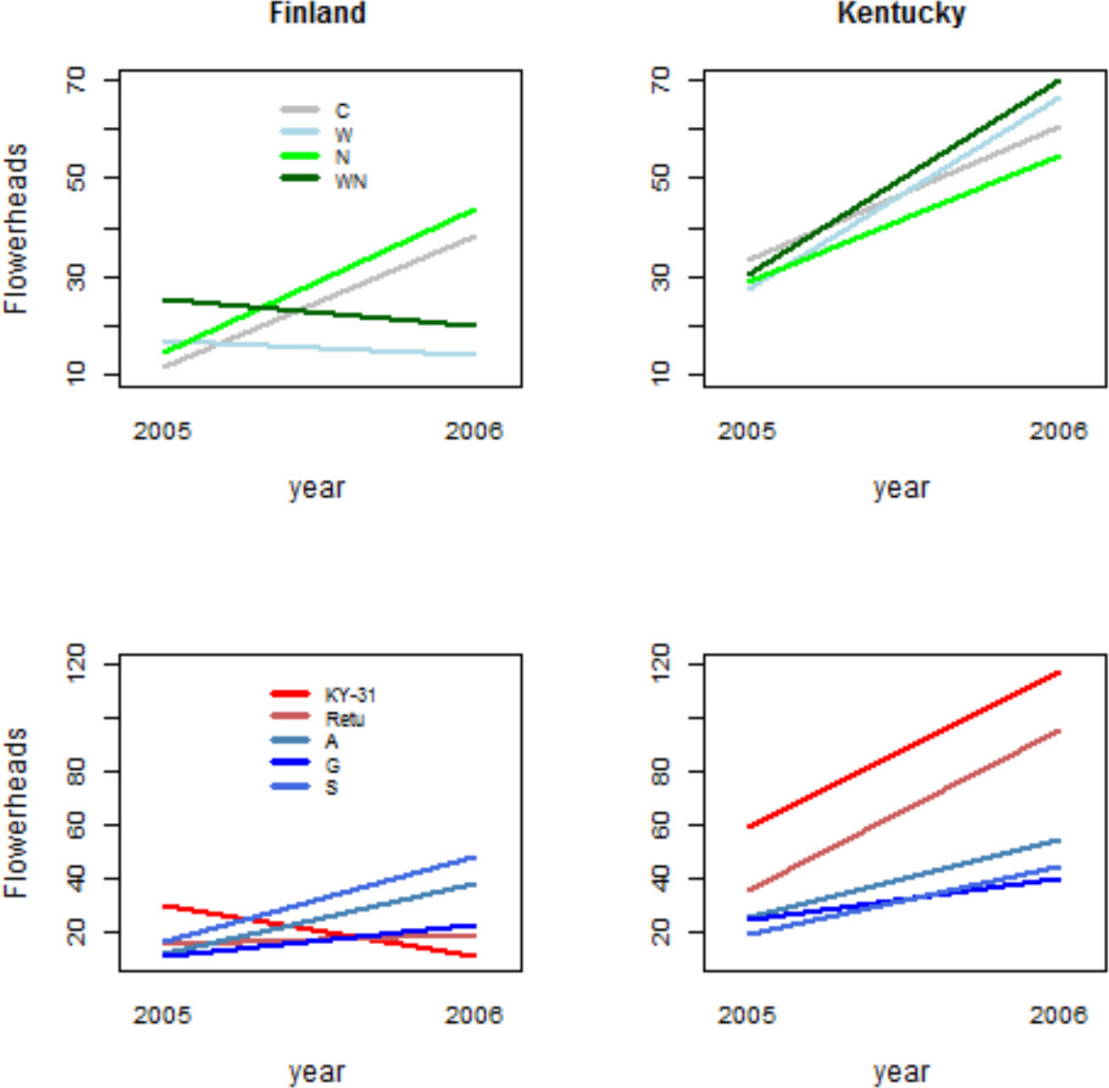
Effects of nutrient and water treatments, and plant origin on tall fescue reproduction. Model-based estimated means of treatments (C = control, W = water treatment, N = nutrient treatment, WN = water and nutrient treatment) and three wild populations and two cultivars (A = Åland island, G = Gotland island, S = coastal Sweden, KY-31 = cultivar ‘Kentucky-31’, Retu = cultivar ‘Retu’) for number of tall fescue flowerheads in Finland and Kentucky experiments in the two study years, 2005 and 2006.

The plants were endophyte infected (E+; the baseline), endophyte free (E-) or manipulatively endophyte free (ME-), and they were given either water (W), nutrient (N) treatments or both (N:W) (Treat; C = control as the baseline). The grasses from the wild origins (Orig) Gotland (G), Åland (A) and Sweden (S) and cultivar ‘Retu’ (R) were compared with cultivar ‘Kentucky 31’ (KY31; the baseline). Results for the same model are shown for both Finland and Kentucky experiment with all main factors and those interactions that were statistically significant in either of the experiments. The main interactions are shown graphically in Fig 5. Model estimates are standard errors (S.E.), degrees of freedom (df), and t-tests (t) with p-values (p) *** < 0.001,** < 0.01,* < 0.05, o < 0.1

### Growth and reproduction in Finland

The plants in Finland were significantly larger in 2005 compared to 2006 (Figs 2 and 3) probably because of the exceptionally dry weather conditions in 2006.

The combined water and nutrient treatment (WN) affected similarly the size of the plants from the three wild European populations (A, G and S) in both study years: the biomass of the wild plants was always highest for WN treated plants (Figs 2 and 4, Table 2). However, the biomass of the two cultivars (KY-31 and R) was not influenced by the treatments, especially in the second study year (Fig 4).

Plant origin did not affect biomass of the plants in the first study year (2005), but in 2006, cultivar KY-31 was the smallest, and cultivar ‘Retu’ the second smallest compared to plants from the European wild populations (Fig 4).

Total flowerhead production was 43% higher in 2006 compared to year 2005. While all wild origins reproduced better in 2006, this differenced could not be detected in cultivars. On the contrary, KY-31 cultivar produced the highest numbers of flowerheads in the first study year (2005), but the lowest number of flowerheads in the year 2006 (Fig 5, Table 3).

The combined nutrient and water treatment affected flowerhead production in the first study year 2005, when WN treated plants produced more flowerheads than the plants in the other treatments. However, in the second study year (2006), C and W treatment plants produced twice as many flowers compared to nutrient treated (N and WN) plants (Figs 2 and 5). This might be partly explained by exceptionally warm and dry summer conditions limiting nutrient cycling of soils and dissolution of applied granular from the top-soils.

### Growth and reproduction in Kentucky

In the Kentucky experiment, plants had 25% higher biomass and produced twice as many flowers in the second study year compared to first study year (Figs 2, 3 and 5).

In the first study year (2005), plants grew slightly, but not statistically significantly, larger on WN treatment plots compared to other treatments (Figs 2 and 4), but produced equal numbers of flowerheads on all the treatments (Figs 2 and 5). In the second study year (2006), biomass of control plants (C) was lowest, whereas biomass in the N and WN treatment was 30–50% higher compared to other treatments (Year*N interaction in Table 2), and the flowerhead production in the WN treatment was higher than in the C and W treatments (Year*Treat N -interaction in Table 3, Fig 5).

Biomass of the two cultivars (Retu and KY-31) was higher compared to wild origin plants (A, G and S) in the first study year (2005), but this difference was not detected in the second study year (2006), when all the plants were about the same size (Year*Orig -interactions for wild origins Table 2, Fig 4). This suggests that the cultivars were less plastic; the biomass was 15–20% and 40–60% higher in 2006 compared to 2005 in cultivars and wild origin plants, respectively. The wild European origin plants grew better under treatments W, N and WN.

Flowerhead production of the two cultivars (KY-31 and Retu) was clearly superior compared to wild origin plants (Table 3). In the second year, the cultivars produced twice as many flowerheads compared to the wild origin plants (Fig 5, Table 3).

### Endophytes

The overall effect of endophyte infection on tall fescue growth and reproduction was significant: E+ grasses tended to be larger and have higher number of flowerheads compared to E- and ME- grasses (Figs 2 and 3, Tables 2 and 3).

## Discussion

Our results support the idea that grass invasions and naturalizations from Europe to North America are more successful than the reverse. All the 480 experimental plants survived in the US, and all the plants of the three wild populations from northern Europe and the Finnish cultivar ‘Retu’ performed well in US throughout the study. In contrast, 9% of the plants died in Finland. Overall mortality was 4% in wild origin plants, 12% in cultivar ‘Retu’ and 18% in cultivar KY-31, being highest in fertilized ‘KY-31’ plants. At the beginning of the experiment KY-31 plants established well in the common garden in Finland. All of them survived the transplantation in 2004 and the first winter, and during the second growing season (2005), their growth was equivalent to and flowerhead production higher than in the other plant origins. However, after the growing season in 2005, their survival and performance declined. During the second study year (2006) in Finland, KY-31 survivors produced lower biomass and fewer flowerheads than the other plant origins. These results suggest that the KY-31 plants are maladapted to high nutrient environments at higher latitudes characterized by harsh winters.

We acknowledge that because these results are from a single tall fescue ecotype KY-31 grown only in two study sites, they should be interpreted cautiously as an indicator of poor invasion success of Northern American origin tall fescue ecotype to Europe in general. However, cultivar KY-31 is a good model to test the invasiveness of tall fescue for the following reasons. First, it occurs from the Pacific Northwest to the southern states and dominates semi-natural grasslands and pastures in the eastern US. KY-31 constitutes majority of all tall fescue which is the most abundant perennial grass in the eastern USA today. Second, it tolerates well a wide range of environmental conditions. Thus, it is regarded as an economical and low maintenance variety in the US. It grows best in moist environments, but its fibrous root system that extends more than one meter deep in soils renders it heat, drought and wear tolerant. Thus, it is perfectly adapted to the climatic "transition zone" of the United States where summers are too hot and humid for most other perennial cool season grasses [28,45]. The adaptive capacity of KY-31 to variable environmental conditions might be related to considerable diversity detected among KY-31 genotypes that may partly be influenced by climatic conditions [46]. For example, in environments characterized by long and harsh winters, juvenile seedlings are prone to winterkill and some genotypes to poor performance. However, well-established seedlings and mature plants are relatively winter hardy. Accordingly, seed companies suggest KY-31 be used also for the northernmost planting zones such as zones 1, 5, 6 and 7 in Maine, Michigan, Minnesota, New Hampshire, New York, Vermont, western Washington and Wisconsin, corresponding to the environmental conditions of the northernmost distribution range of the species in Europe in terms of seasonal changes in temperatures. High establishment success, and growth and flowering during the first growing season in our study suggest that KY-31 should be equally well adapted to endure winters in southernmost Finland as the other Continental morphotype tall fescue germplasms. Finally, as the oldest, most widely planted and successful tall fescue cultivars, it has relatively long adaptive history in the US. KY-31 was developed from only a few plants from a population adapted to the local seasonal changes in day length, severe drought and heavy vertebrate grazing [28,32]. This selective bottleneck and founder effect was largely responsible for KY-31 traits that may have made it successful in the US but not in Europe. Thus it is a good model to test whether the selection for adaptive characters for success in North America has limited the species invasiveness back to Europe.

Overall in the Kentucky experiment, the biomass and flowerhead production of KY-31 plants were 9% and 47% higher compared to other plant origins in the end of the second growing season (2006). Similarly, the Finnish cultivar ‘Retu’ plants produced nearly comparable number of flowerheads to KY-31 plants. Furthermore, the higher number of flowerheads in KY-31 and ‘Retu’ plants relative to wild plants from Europe was consistent across all the nutrient and water treatments. In contrast, the biomass production and flowerhead production of the wild plants was highly dependent on nutrient and water availability in soils. This suggests that tall fescue performance in the US largely depends on environmental conditions and plant origin, and wild plants are phenotypically more plastic than cultivars.

Our results do not strongly support the hypothesis that endophyte invariably confers an invasiveness advantage over uninfected plants. Overall biomass and flowerhead production of E+ plants were higher compared to E- and ME- plants both in the US and Finland, suggesting that the endophyte might confer invasiveness advantage over uninfected plants. However, the advantages from endophytes appear to be highly context dependent. In many cases, environmental conditions and plant origin appear to override the effects of endophyte, and plant responses varied among years. For example, E+ plants originally collected from Åland islands grew significantly bigger and produced higher number of flowerheads than their endophyte-free counterparts in Kentucky, particularly in the second growing season (2006). However, much of this enhanced plant growth was caused by watering and fertilization treatments. Furthermore, regardless of endophyte infection, KY-31 performed poorly in Finland, and the endophyte-free Finnish cultivar ‘Retu’ appears to be well adapted to wide range of environmental conditions, including those in the US. These results suggest that the fungus and host individually or in concert as a phenotypic unit, respond to local selection pressures, and the adaptations of plant or the symbiotum to their original environments largely determine their responses to new environments.

Our results do suggest, however, that the endophyte symbiosis can modulate adaptive traits of tall fescue. Overall biomass and flowerhead production was highest in E+ and lowest in ME- plants, but the positive and negative effects of symbiotic endophyte and its removal varied geographically and between the plant traits. The poor performance of ME- was particularly pronounced in terms of biomass production in Finland. This suggest that phenotypic selection operates on plant-microbe symbiotum, and in some cases the loss of fungal partner may lead to constrained adaptive capacity in the host grass [26]. This may be related to “compensated trait loss”, i.e., some plant functions may have been lost when they are instead provisioned by the interacting fungal partner during the long coevolutionary history of the endophyte and the host grass [26,47]. Therefore, the loss of infection, and the consequent decrease in plant growth and reproduction, and possibly resistance to herbivores, may have similar or even greater importance for the species’ range limits than the positive effects of endophytes to the adaptive grass traits.

Our results demonstrate that all the plants of higher latitude origin performed well when transplanted in the south, whereas KY-31 performed poorly when transplanted to the north, supporting the idea that local adaptations may limit the range shift of tall fescue polewards. Temperature presumably determines overwintering and thus the northernmost distribution range of the ecotypes of the species. Thus, different tolerance of KY-31 to colder winters of the northernmost distribution range of the species may partly explain these results. However, both study sites are characterized by seasonal fluctuation in temperature, including periodic frost, and the KY-31 is successfully grown in colder climatic conditions north from the study site in the US. Furthermore, our results show that the possible range-limiting temperature tolerance of KY-31 is interactively affected by ontogeny of the plants and other environmental forces. Harsh winters caused some plant deaths in all plant origins in Finland, but only two plants died in the first winter and the mortality in the subsequent winters was particularly pronounced in KY-31 plants and only in fertilized soils. These results demonstrate that winter temperatures do not limit the establishment and survival of KY-31 more than European origin germplasms in low nutrient environments. In contrast, better growth conditions in terms of nutrient rich soils appear to decrease plant tolerance to winter temperatures. For example, the ability of KY-31 to allocate higher proportion of resources to aboveground biomass than to roots could explain the higher mortality of KY-31 compared to other origins.

We hypothesize that these results were in part due to different adaptations of plants to seasonal changes in day length and light quality when they were transplanted across latitudes. The striking difference between the study sites in the US and Finland is a shorter growing season in the Finnish site situated at higher latitude, and associated stronger photoperiodism (S3 Fig). The optimal timing of growth, reproduction and adaptations related to winter hardiness such as resource allocation to storage organs are critical to tall fescue fitness in both locations of the cross-latitudinal transplant experiments. However, the consequences of phenological mistiming in the plants are presumably more critical when plants are transplanted to higher latitudes. From other studies, both temperature and seasonal changes in day length and light quality are known to be central factors regulating and coordinating tall fescue seed germination, growth, reproduction and development [48–50]. We propose that although the European origin tall fescues that are adapted to photoperiodic cues in northern latitude may have limited ability to fully exploit the longer favorable growing season in lower latitudes, the optimal timing of growth, reproduction and adaptations related to winter hardiness such as resource allocation to storage organs are more critical to plant fitness in higher latitudes where the favorable growing season is shorter [4].

Our results have broader importance for understanding the expansion potential of species across latitudes. Our findings suggest that tall fescue invasions from higher latitudes to lower latitudes are more successful than the reverse. We propose that adaptations to seasonal changes in photoperiod in combination with cold winters play the key role causing fatal mistiming in the phenology of tall fescues transplanted to higher latitudes. To refute or support this hypothesis will be left to the future studies explicitly testing the phenological events of tall fescues in reciprocal transplantation experiments across latitudes. In particular, these studies should take into account that in natural populations, selective forces are more variable and can operate simultaneously on several traits, or plasticity in traits, of the fungus, host or host–fungus unit. In contrast to decreased fitness of the KY-31 plants in higher latitudes, our results showed that all northern origin tall fescue germplasms, regardless of their endophyte infection status, performed well when transplanted to lower latitudes. This suggests high potential of Eurasian germplams in agriculture in the US, and should be taken into account in tall fescue breeding programmes.

## Supporting Information

S1 Data Biomass and number of flowerheads of the experimental plants. (XLS)

S1 Fig Growth and reproduction of tall fescue. Biomass (x±S.E.) and number of flowerheads (x±S.E.) of wild origin (A = island of Åland, G = Island of Gotland, S = coastal Sweden) and cultivar (Retu and KY-31) plants treated with water (W), nutrients (N) or their combination (WN). C = control with no water or nutrient applications.
(PDF)

S2 Fig Seasonal changes in day length at the study sites. (PDF)

S3 Fig The experimental field at the University of Kentucky experimental farm in Eden Shale, Kentucky, USA. (JPG)

S4 Fig The experimental field at Turku Botanical Garden, University of Turku, Finland. (JPG)

S1 Table Monthly rainfall and mean temperature at the study sites in Kentucky and Finland. (PDF)
