# Supplementary figures and images for: Performance of Endophyte Infected Tall Fescue in Europe and North America
Source: PLoS One. 2016 Jun 10;11(6):e0157382. doi: 10.1371/journal.pone.0157382 (PMC4902185; doi:10.1371/journal.pone.0157382)

**Finland 2005**

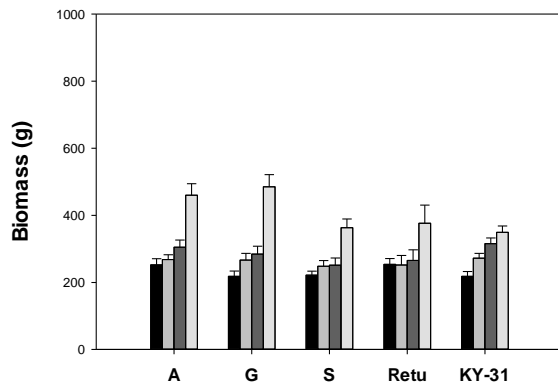

**Finland 2006**

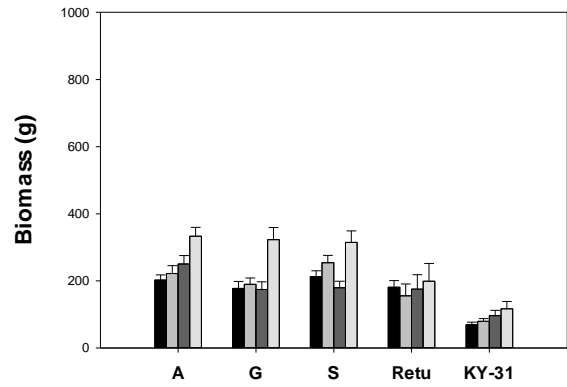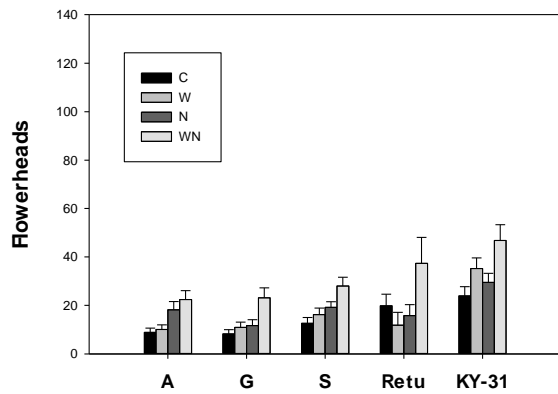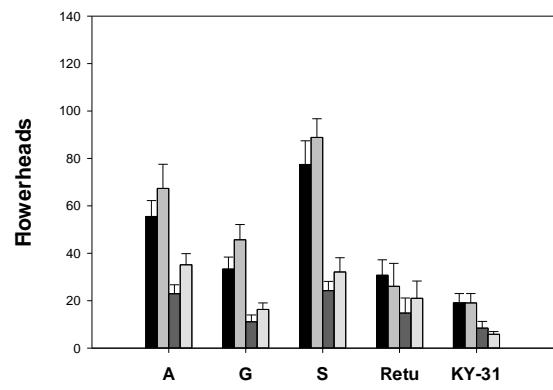

**Kentucky 2005**

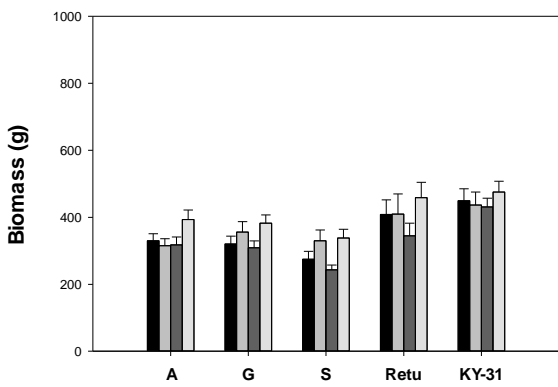

**Kentucky 2006**

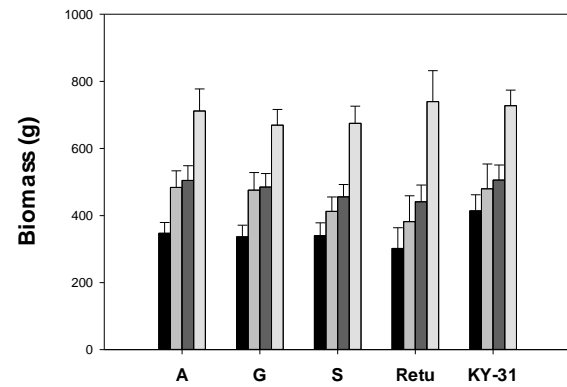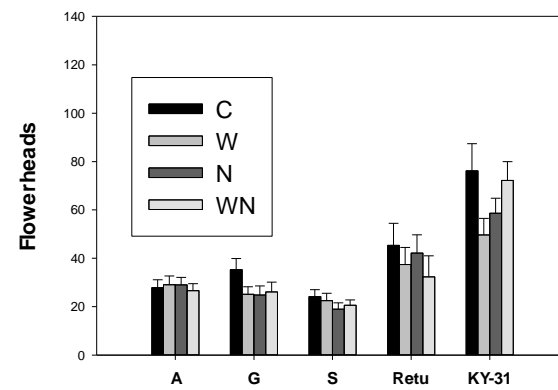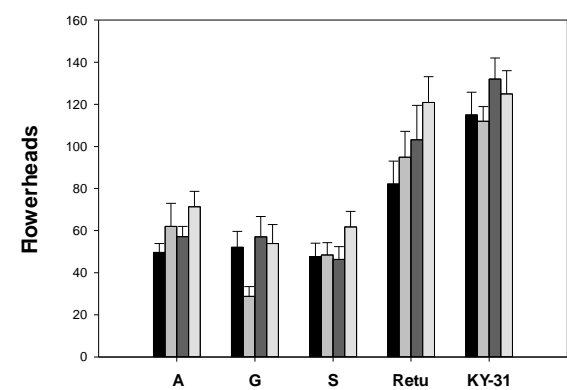

Supplement: S1 Fig — Biomass (x±S.E.) and number of flowerheads (x±S.E.) of wild origin (A = island of Åland, G = Island of Gotland, S = coastal Sweden) and cultivar (Retu and KY-31) plants treated with water (W), nutrients (N) or their combination (WN). C = control with no water or nutrient applications. (PDF) [file pone.0157382.s002.pdf]

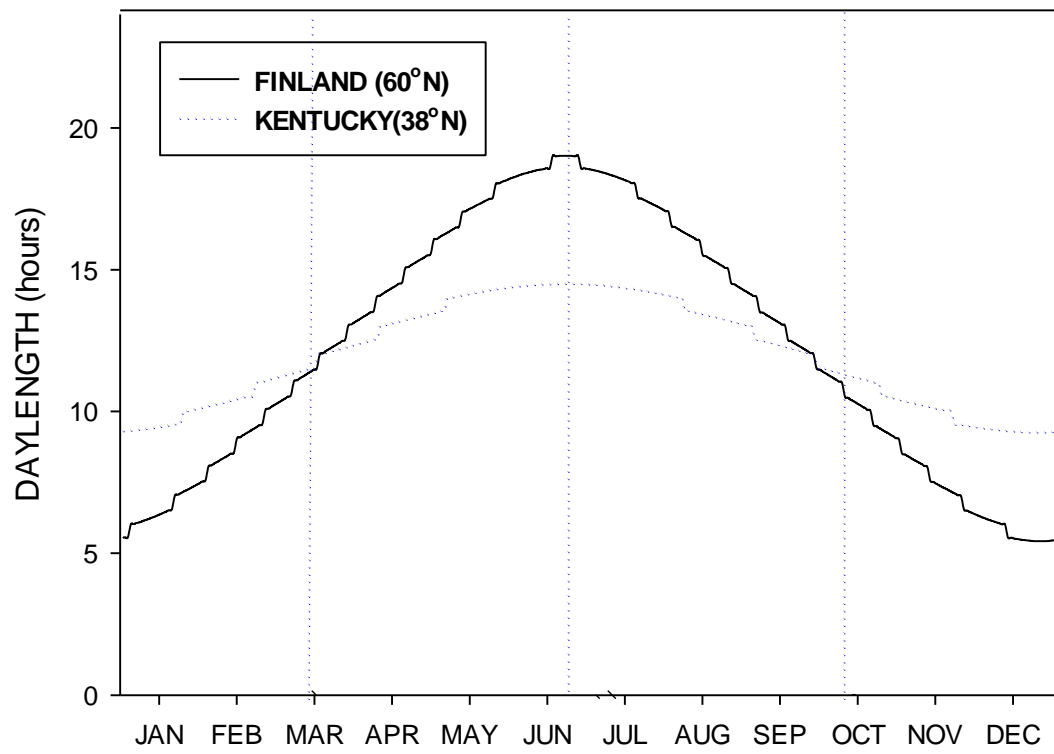

Supplement: S2 Fig — (PDF) [file pone.0157382.s003.pdf]

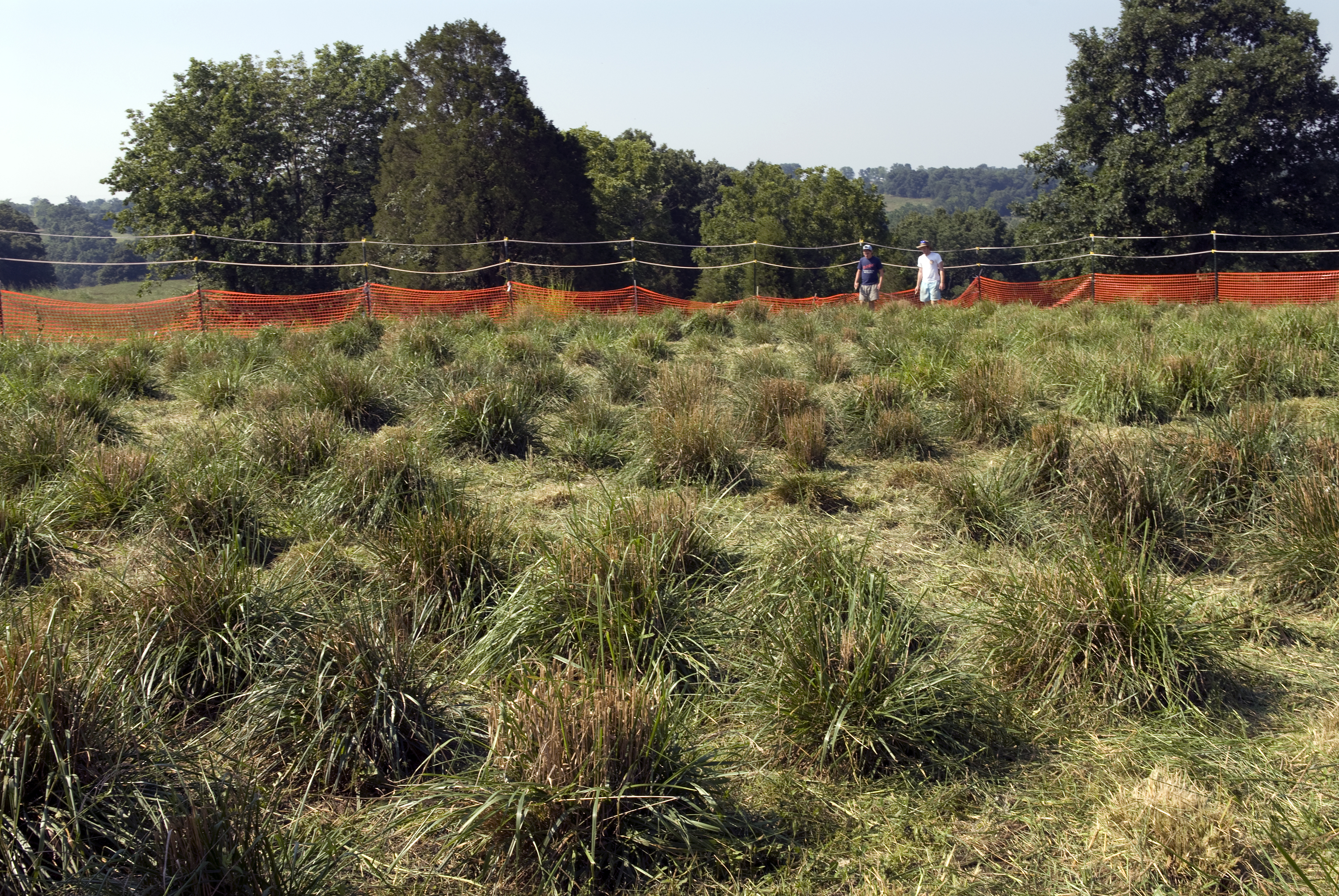

Supplement: S3 Fig — (JPG) [file pone.0157382.s004.jpg]

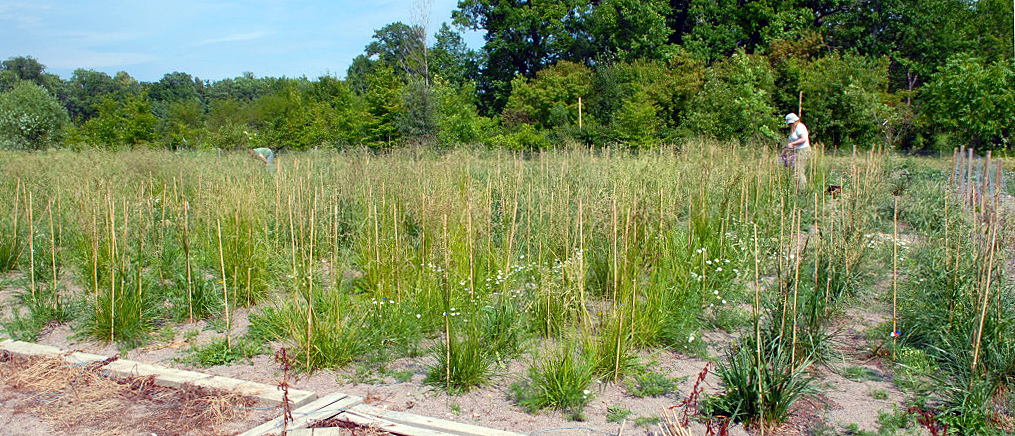

Supplement: S4 Fig — (JPG) [file pone.0157382.s005.JPG]
