# Supplementary material for: Performance of Endophyte Infected Tall Fescue in Europe and North America
Source: PLoS One. 2016 Jun 10;11(6):e0157382. doi: 10.1371/journal.pone.0157382 (PMC4902185; doi:10.1371/journal.pone.0157382)
Supplement: S1 Table — (PDF) [file pone.0157382.s006.pdf]

	Year	Jan	Feb	Mar	Apr	May	Jun	Jul	Aug	Sep	Oct	Nov	Dec
FINLAND													
Temperature C°	2005	-0.6	-4.4	-5.5	4.6	9.3	13.8	18.8	16.0	12.2	7.4	3.4	-3.7
	2006	-4.0	-7.0	-6.0	3.5	10.4	14.9	18.1	18.2	13.8	7.6	2.6	3.8
	2007	-2.1	-8.7	2.4	4.6	10.5	15.7						
Rain mm	2005	104	40	4	16	35	38	144	136	35	49	102	37
	2006	33	19	24	55	35	40	63	48	34	168	112	85
	2007	91	7	34	26	69	74						
KENTUCKY													
Temperature C°	2005	1.6	3.7	4.3	13.3	15.9	23.6	25.3	25.5	21.7	14.1	8.1	-0.5
	2006	5.4	1.4	6.4	14.7	16.5	20.3	24.8	25.1	17.9	11.9	8.1	5.1
	2007	2.2	-3.5	10.5	11.2	19.8	23.6						
Rain mm	2005	144	52	96	93	53	49	82	218	40	44	92	71
	2006	141	52	157	133	91	129	112	97	219	151	43	94
	2007	95	88	137	150	19	57						
